# Highlights on Cerebral Arteriovenous Malformation Treatment Using Combined Embolization and Stereotactic Radiosurgery: Why Outcomes are Controversial?

**DOI:** 10.7759/cureus.1266

**Published:** 2017-05-22

**Authors:** Faustina N A Sackey, Nathaneal R Pinsker, Benjamin N Baako

**Affiliations:** 1 Loeb Health Research Institute at Ottawa Hospital, University of Ottawa, Ontario, Canada; 2 Internal Medicine, Kings College Hospital; 3 Surgery, University of Ghana School of Medicine and Dentistry

**Keywords:** arteriovenous malformations (avms), controversial outcomes, combined embolization and stereotactic radiosurgery (embo/srs), setbacks

## Abstract

Cerebral arteriovenous malformations (AVMs) are abnormal tangling between brain arteries and veins causing an arteriovenous shunt called nidus with an intervening network of vessels from the region of formation and spans through the brain. AVM effect is debilitating to the affected individual due to associated persistent intracerebral hemorrhage, resulting in significant occurrences of seizures and neurological damage. Recent innovative treatments involve a combination of embolization (Embo) procedures followed by stereotactic radiosurgery (SRS), designed to optimize less-invasive practice for the obliteration of the AVMs. Three groups of investigators reported different outcomes based on obliteration rates and adverse events, making the effectiveness of options for therapy, controversial. We have taken the case-oriented-approach to highlight on varying outcomes from various studies and provide insights as to why findings from different operation settings could be so conflicting.

We chose 18 articles for systematic analysis based on initial electronic database selection of 40 key papers already identified for inclusion, followed by independent blinding assessment by two co-authors. Our evaluation was based first on our specific inclusion criteria, examining method quality, obliteration rates, serious adverse events (SAEs) and mortality rates. Second, we made a comparison between SRS or embo alone treatments versus combined embo/SRS procedures, relative to AVM sizes, following Spetzler-Martin (SM) method. Third, we considered publications which had concrete statistics with well-defined P-values and clarified outcomes for accurate evaluation.

We found that patients with small to medium-sized AVM were susceptible to either embo alone or SRS alone treatments, yielding obliteration rates from 71%-100%. Except for one report, giant sizes AVMs were not amenable to these single treatments, subjecting patients to embo/SRS procedures, which yielded mixed results: One group reported 52%-65% obliteration rates, compared to 23%-28% embo alone treatment. A second group contradicted this apparent beneficial outcome, obtaining obliteration rates of 53% with combined treatment compared to 71% with SRS alone, four-year postoperative. A third group reported there was no difference between single and combined treatments and obtained complete obliteration of 70%-82%, ranging from three-five-years postoperative follow-up. In all the cases analyzed, obliteration rates improved with time. SAEs, such as persistent hemorrhage and permanent neurologic deficits (P-NDs), as well as mortality, were minimal during intraoperative and postoperative follow-ups.

The problem of conflicting outcomes in combined treatments of AVM by EMBO/SRS exists. Previous investigators, however, have overlooked to address this issue satisfactorily. Our analysis found that the reported inconsistencies in AVM treatment outcomes are attributable to key factors making therapy unpredictable, which includes: the size of the AVM, nidus localization and accessibility of either Embo or radiation dose applied, certain Embo materials lowering obliteration rates by masking radioactive effect on the nidus during SRS and follow-up timing for obtaining obliteration rates determine the extent of obliteration.

We have indicated critical factors which require consideration when planning strategies for treatment of AVM patients and have made suggestions of how to overcome such hurdles.

## Introduction and background

Cerebral arteriovenous malformations (AVMs) are irregular connections of the brain capillary network [1-3]. Typically, AVMs localize in the cerebral hemisphere as cone-shaped lacerations with the apex of the cone approaching the ventricles [3-4]. According to the American Heart Association, cerebral AVM is a disorder which occurs in approximately two to five in 1,000 individuals in a general population, and it is more common in males than females [3-4]. Diagnosis is prevalent in the third decade (30 years) of life during which intracranial hemorrhage or seizure is frequent [3-4]. About 50% of the hemorrhagic cases results in 53%-81% morbidity and 10-30% mortality [3-4].

The etiology of AVM formation is not clear; nonetheless, it has been postulated that genetic factors involving gene alterations with subsequent overexpression or hyperactivity of blood flow hemodynamic regulatory elements, as well as, vascular endothelial growth factor (VEGF-A) signaling [1] might play roles. Additionally, epigenetic factors might act by modifying the activity of vascular controlling genes via deoxyribonucleic acid (DNA) methylation [5]. The consequences of these alterations are enhanced and progressive vascular drainage malformation, which leads to frequent hemorrhage encountered by patients are affected by brain AVMs. Recent endeavors to map the molecular basis of the underlying cause of the disorder are gene linkage and genetic mutation analysis of the defective hemodynamic controlling factors [6].

Although AVM development can occur anywhere in the body, cerebral AVMs tend to be most debilitating, especially, if they reside in eloquent brain regions which are parts of the brain controlling speech, mobility and senses. AVMs in eloquent locations can rupture easily and cause intracranial hemorrhage, thus, presenting symptoms such as seizures, stroke, impaired speech, and vision, broadly classified as neurological deficits [7-9]. Other notable symptoms are paralysis, loss of coordination, mental disorder, memory depredation (dementia), hypoxia, severe headache, feeling of numbness and dizziness [7-9]. Therefore, the cause of treatment is to alleviate the risk of high-pressure blood flow through these malformed vessels to prevent vessel rupture and improve or preserve neurological function.

AVM is diagnosed by the use of computed tomography (CT) scan, magnetic resonance imaging (MRI) and cerebral angiography which involves X-radiation (x-ray) imaging, following contrast injection [9-11]. Briefly, the dye is injected into a catheter inserted in the patients’ femoral artery which then travels through the neck internal carotid artery to the brain. Pictures are taken to reveal the localized position of the AVM. These imaging tests are important in that, apart from showing the location of the AVM, they can be used to identify the original size, change in size after treatment, and whether the AVM had been bleeding or unruptured. Typically, estimation of the AVM nidus is carried out by using a scoring method called Spetzler-Martin (SM) grading scale [12], which helps in predicting the risk of surgical mortality and serious adverse effects (SAEs). SM grading scale puts into consideration; a) nidus size and b) eloquent localization of the AVM, that is, the parts of the brain that control speech, motor functions, and senses known as the brain cortex. The latter includes the brainstem, thalamus, hypothalamus and the connection between the cerebellum and the brain stem called cerebellar peduncles, as well as, visible and language regions, and c) the existence of deep venous drainage. Nidus sizes less than three cm, three-six cm, and less than six cm are scored one, two and three points, respectively. AVM in the eloquent region is scored one point, while the existence of deep vein drainage scores one point. Thus, a patient with AVM total scores of six points has a prognosis that scores all the points that ultimately lead to SAEs and thus is predicted to be at most mortality risk on surgical outcome. Health conditions may also play roles in determining surgical outcomes even of patients with lower SM grading scores [13]. We reviewed a set of articles to address controversies in reported outcomes, the probable causes and suggested cautionary notes for remedy towards successful treatment outcomes.

## Review

### Search strategy and articles selection

Between December 2015-2016, we searched the medical literature analysis and retrieval system online (MEDLINE) database via PubMed, Excerpta Medica database (Embase) Database-Ovid, and Elton B. Stephens Co. (EBSCO’s) Medical Database for brain atrioventricular malformations and intracranial AVMs ranging from 1995 to 2016. We supplemented our searches with Google search engine on the same topic. The keywords used were as follows: brain AVMs, radiosurgical treatments, stereotactic radiosurgery (SRS), embolization (embo), combined embo/SRS, small AVMs, large AVMs, treatment outcomes.

We obtained a total of 40 abstracts from the searches, out of which we chose 30 relevant full-text articles. Two reviewers who mutually agreed on inclusion/exclusion selection criteria screened the reports. Co-author one selected 20 articles (first set). Co-author two blinded to the first co-author, evaluated the 30 papers independently, screened and also selected 20 articles (second set). The two co-authors held a mutual consent meeting and selected 18 articles which appeared in both sets of selections to be included in the study and resolved any discrepancies to avoid observer bias in articles selection.

### Study selection

*Inclusion Criteria:* Our inclusion criteria considered the following: 1) In-depth description of AVM surgery methodology. 2) Large patient population size (>20-2,000). 3) Single SRS and combined embo/SRS treatments and outcomes. 3) AVM sizes and grouping by SM grading. 4) Serious adverse events (SAEs). 5) Obliteration rates. 6) Comparable statistical analysis. 7) How often the authors’ published in the field (to ensure that treatment strategies and methods had been refined by experienced investigators for data collection, analysis, and interpretation). 8) Case mortality. 9) Routine follow-up.

*Exclusion Criteria:* Our study excluded the following criteria: 1) Non-brain AVM. 2) Conventional micro-neurosurgery. 3) Small patient population size. 4) Lack of in-depth description of methodologies. 5) Embo alone treatment/no SRS. 6) Invasive surgery or invasive neurosurgery. 7) No detailed statistical analysis (to be certain that the data we gather would have clear explanations of standard deviations, comparisons within and between groups, and sound statistical significance measurements for reliability). 8) Greater than 20% patient dropped out from follow-up.

### Determination of study characteristics

We assigned the following features in the articles and made comparisons in our assessment: 1) Treatment of large AVM sizes. 2) Obliteration rates (%). 3) Years of study conduction. 4) Patients with brain hemorrhage (%). 5) Permanent Neurologic deficits (P-ND) (%). 6) Mortality rates (%).

### Data extraction, analysis, and presentation of results

We synthesized data from the articles, described the studies narratively and tabulated study characteristics. Presented in the textual format are results which are consistent with the data submitted. Thus, 18 AVM treatment full-length articles were selected, of which six contained single SRS or SRS, as well as, combined embo/SRS treatments, and 10 contained combined Embo/SRS alone treatments. We compared outcomes and pulled results which were similar and those that were contradictory or were not significantly different. Subsequently, a systematic review was carried out to assess methodological quality and outcomes.

We excluded reports with small patient numbers in AVM combined embolization/SRS treatments to ensure confidence level (95%-99%) of a large number of patients were used to generate reliable outcome data. Since the issues, we were addressing were varied outcomes from different reports, it was critical to ensure that the outcome data we were comparing were generated with precision.

Although we used obliteration rates, percent hemorrhage, and permanent neurological deficits (P-NDs) data from previous studies, all of the three authors examined the data from the articles and came to data agreement with reported numerical values via inter- and intra-observer angiography reviews. We subsequently confirmed obliteration rates, percent hemorrhage, and permanent neurological deficit (P-NDs) values reported in the articles. In an instant where there was a lack of quantitative obliteration rate, we averaged our estimation from the angiography and used the mean value. We found trends that might resolve issues of conflicting results, addressed later in this section.

### Techniques used previously in determining obliteration rates and serious adverse events in the articles evaluated

The AVM treatment techniques involve passing a tiny catheter into the cerebral vessels feeding it until it reaches the nidus [9]. Embolization material is then injected to occlude most of the AVM to make subsequent stereotactic radiosurgery (SRS) removal feasible and safer by reducing operation time associated with blood loss during the resection of the lesions [13-14]. SRS procedure entails passing a beam of radiation to target the lesion. Over approximately two-three years of patient follow-up routines, it is possible to track the time course of obliteration of the AVM nidus. Outcomes were analyzed by comparison of digital subtraction angiographies (DSA) before and after treatment, as well as, clinical presentation results [15-20]. Briefly, obliteration rates were measured by obtaining ratios between the original nidus size and follow-up periods after treatments, and also, morphology evaluation of restricted venous outflow. Seizures were measured by angiography results of venous drainage patterns. The extent of hemorrhage was measured by determining velocity and volume flow rate (VFR) by angiography.

Statistical methods typically used to quantitate outcomes are univariate (T-test, chi-square, Fisher’s exact test) and multivariate Cox-regression or logistic analyses [9,14,7], the use of the log-rank test [13] in risk factor determination characterized by obliteration, hemorrhage, radiation-induced changes and post radiosurgery adverse events [8,14-19]. Obliteration rates are also determined using Kaplan-Meier analysis. Comparison between study groups is made using Fisher exact, chi-square or Mann–Whitney–Wilcoxon test [8,14-19], having a P-value < 0.05 indicating statistical significance. Flickinger Pollock scale [20-21] is used to test for variable continuity and frequency categorization.

### AVM treatment procedures employed in the articles evaluated

AVMs sizes were determined using the Spetzler-Martin (SM) grading method [12] or by volume measurements [22] after DSA, before making a decision on treatment strategy. Whether or not treatment can be made possible is dependent on the AVM size, it's possible location, patient age, and health history. For example, if the volume of a patient nidus is SM V-VI and localized in eloquent regions, the risk of SAEs will carry more weight than treatment, and a decision could be made to leave the nidus untouched. For smaller AVM sizes (SM I, II and at times, III), microsurgical resection, also known as stereotactic radiosurgery (SRS), has been the profound treatment choice with significantly high obliteration rates (mean, 50%-68%) [15-19]. Certain investigators have also reported the use of embolizers such as Polyvinyl alcohol (PVA), n-butyl cyanoacrylate (nBCA), recently, Glubran 2 and Onyx [23], which all tend to shrink the AVM, curing low percentage of patients, but in the majority of cases, low obliteration rate have been the outcome for embolization (Embo) alone treatment. Contrary to promising results for single micro or radiosurgical procedures, patient treatment has been more challenging with AVMs which have SM grading sizes >III-VI [14,24-25]. Because of their large size, the AVMs are tough to uproot by single SRS procedure, and repetitive SRS sessions always produced disabling outcomes, compared to single operations [26]. Hence, the adopted strategy is to utilize a combined multidisciplinary therapy of initial embo procedure to reduce the size of the nidus [9, 14-18] before SRS, using gamma knife (GK) [14,25-29], or Linear Accelerator (LINAC) [27-28]. Embo also minimizes SRS-related complications in the majority of cases [9, 14-17,22-25]. Therefore, it has become an adjunctive tool used before SRS procedures for treatment of large masses. The aim is to eradicate the AVMs with high efficacy while maintaining healthy tissue surrounding the mass. Unfortunately, using combined embo/SRSg for treatment and management of AVMs have yielded different outcomes and thus remain controversial [14-17, 30-33, 34-36]. To address the reasons behind these differences, a set of articles were selected and analyzed, as discussed below.

### AVM size and timing play roles in controversial outcomes according to most reported cases

Betramello, et al. [29] reported 255 out of 277 patients with cerebral AVMs treated cases over a five-year period (2000-2004) in the neurological facility. Ninety-eight underwent combined embolization with polybutyl cyanoacrylate (PBCA), Glubran two, Onyx or Glubran two/Onyx, and a single GK treatment; 16 patients went through embolization alone treatment, while 113 had single SRS treatment because of their small nidus size (results not reported). Out of the 98 patients that underwent combined treatment, 44 completed the follow-up routine. The clinical parameters measured included hemorrhage, epilepsy, focal neurological defects and cognitive impairment. Shown in Table 1 is a summary of the results [29].

**Table 1:** Summary of outcomes of arteriovenous malformations (AVM) patients treated with single embolization (embo) or combined embo/stereotactic radiosurgery (SRS)

Characteristics	Combined Treatment (Embo + SRS)	Single Treatment (Embo)
Number	44	16
Total obliteration	23 (52.3%)	4 (25.0%)
Hemorrhage	14 (31.8%)	2 (12.5%)
Epilepsy	16 (36.4%)	1 (6.25%)
Focal deficit	2 (4.5%)	1 (6.25%)
Technical complications (glued catheter)	4 (9%)	0
Mortality	0	0
Non-eloquent nidus location	18 (40.9%)	2 (12.5%)
Eloquent nidus location	22 (50%)	2 (12.5%)
Nidus volume <10 cm^3^	19 (44%)	4 (25%)
Nidus volume 10-20 cm^3^	15 (34.1%)	0
Nidus volume >20 cm^3^	6 (13.6%)	0

**Figure 1:**
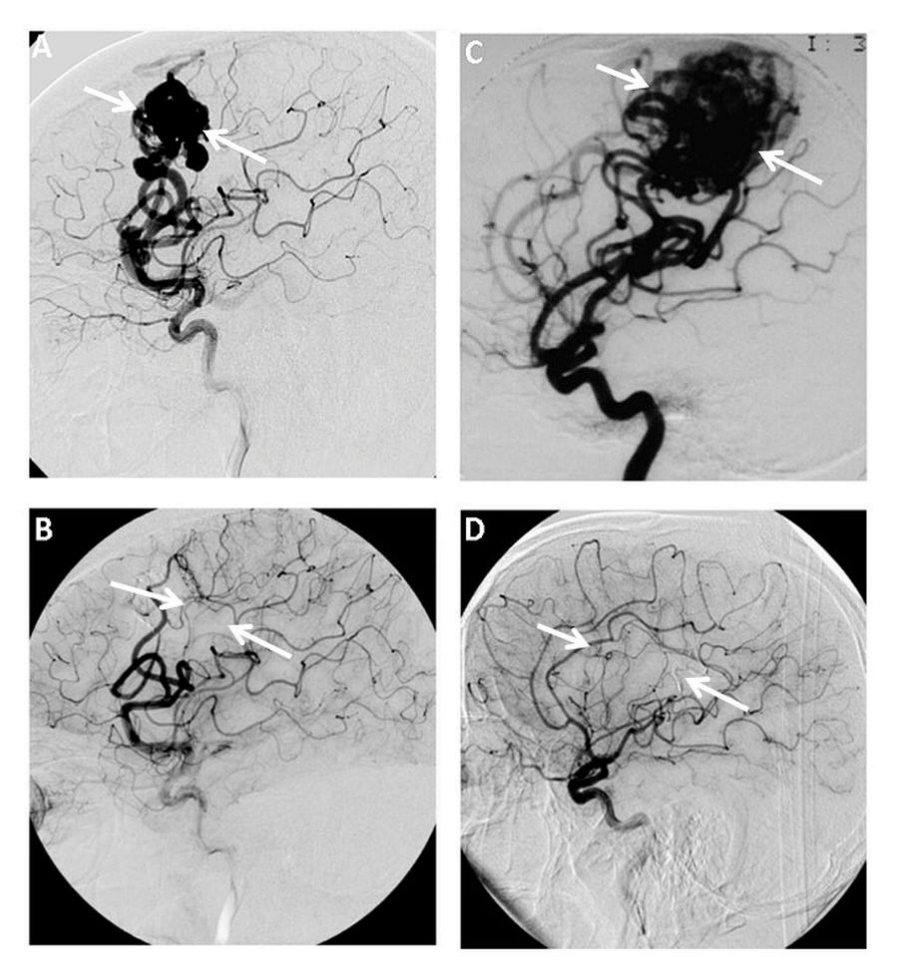
Angiography images showing changes in arteriovenous malformations (AVM) obliteration rates before and after combined treatment over a time (A) Small arteriovenous malformations (AVM) size obtained before treatment and (B) immediately after Onyx embolization with massive AVM size (C) taken before treatment, and (D) after three years follow-up after combined embolization/SRS treatment. (AB) Pre-rolandic digital subtraction angiography (DSA) of a 60-years-old male with nine cm^3^ AVM volume, presented with aplasia. Images were taken before (A) and immediately after Onyx embolization (B), showing complete obliteration of the AVM nidus. (CD) Right rolandic DSA images from a 42-years-old male with 22 cm^3^ AVM volume, presented with a partial seizure. Before treatment (C) and three-year follow-up after combined treatment with Onyx/SRS (D), also showing complete obliteration of the AVM nidus. White arrows indicate the position of the AVM. Figure reproduced from Betramello, et al. [29]

As shown in the Betramello, et al. study [29], the purpose of the combined treatment was to be able to treat AVMs with gigantic SM grades. The results precisely showed the combined treatment worked better with respect to reducing the size of the AVM (52.3% obliteration rate), compared to the embolization treatment alone (25%). There were more temporal SAEs with the combined treatment, but this could have been attributable to the aggressive nature of the treatment. The single SRS treatment outcomes were not shown to allow comparison. Shown in Figure 1 is an interesting observation; the DSA images revealed before (Figure 1A) and after administering a single Onyx treatment to a 60-year-old male with nine cm^3^ AVM size. He experienced an instant complete obliteration (Figure 1B). In a like manner, a 42-year-old male with a much larger AVM size of 22 cm^3^ before treatment (Figure 1C), also displayed the same obliteration outcome, but over a much longer period. Despite the time factor involved, he had five successive embolization procedures followed by a single SRS. Further, he went through a follow-up period of three years (Figure 1D).

**Figure 2:**
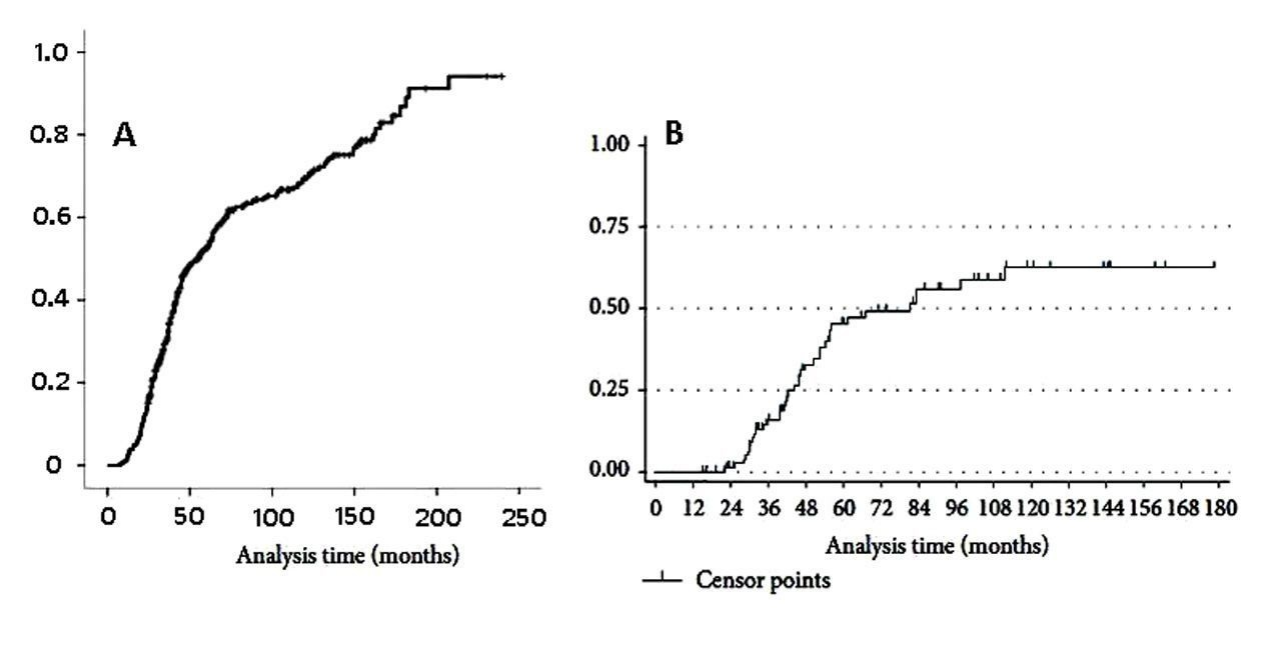
Plots showing obliteration rates of AVM, versus time, after single stereotactic radiosurgery (SRS) or combined embolization/SRS treatments (A) Time course of cumulation of obliteration rate obtained from 444 patients with AVM largely of Spetzler-Martin (SM) III-IV sizes who underwent 20 Gy SRS alone treatment with 20 years follow-up period. (B) A parallel comparative graph from a study by another group but with 95 patients with approximately 50% of the group presented with giant size AVMs (SM IV-V) in eloquent regions who underwent a combined therapy sessions of Onyx embolization and SRS treatments with 15 years follow-up period. Figures reproduced from Ding, et al. [15] and Dalyai, et al. [14]

The results suggest that achievement of an effective obliteration with a much larger AVM requires stepwise sessions, aggressiveness, and time-effective treatments. Thus, investigators need to consider these factors as contributing to an overall consistent outcome when grouping patients in a study. Similarly, Ding, et al. [15] reported obliteration rates obtained from 72 patients diagnosed with AVM by MRI or DSA. These patients (44.5%) had at least AVM sizes not less than SM III. They underwent single SRS with 20 Gy dose and follow–up monitored by determination of obliteration rates obtained by data analysis from series of DSA images and plotting a graph from the results, as shown in Figure 2A. This plot parallels with obliteration time course obtained by another group, Dalyai, et al. [14] in figure 2B, who instead of a single SRS treatment, performed a combined N-butyl cyanoacrylate (nBCA) embolization and 21 Gy gamma knife surgery (GKS) procedures in 95 patients of which 47% had large AVM, SM IV-SM V.

In contrast to the graph obtained by Ding and his group, obliteration rate in the latter was obviously slower and appeared to be reaching its peak with approximately 52% rate around the fifth year (Graph B). Graph A, on the other hand, went through a second phase after the seventh year and the obliteration rate reached about 95% by 18 years. It seems from this analysis that the single SRS treatment has resulted in a better obliteration outcome than the combined treatment. The inference likely to be made from these two reports is that the SM sizes of the AVMs in both studies vary and so the comparison is being drawn between small sizes AVM in (Figure 2A) and large complex AVM sizes (Figure 2B) and that the latter had imposed limitations on the obliteration outcome. Large AVM sizes once more reflect on the requirements for more aggressive treatments such as stepwise embolization sessions, increasing the linear accelerator (LINAC) or the gamma knife (GK) radiation dosage, as well as a requirement for much longer follow-up period to achieve significant or complete obliteration.

The obliteration rates obtained by Dalyai and co-workers were lower than those reported by other investigators, which also reflects the large AVM sizes among 47% of their patients (SM IV-V) and the initial mean volumes, which was 22 cm^3^. These are obviously very complex and gigantic malformations. Thus the purpose for embolization with nBCA before SRS was to generate a combination of effects for an aggressive treatment which produced 63% obliteration rate by 10 years (Table 2) [14].

**Table 2:** Summary of outcomes of AVM patients treated with combined embolization/stereotactic radiosurgery (embo/SRS) over a 10-year follow-up period

Characteristics	1-3 years 5 years 7 years 10 years			
Total obliteration rate	40%	45%	56%	63%
Hemorrhage	26 (27%) of 95 patients			
Procedure complications	14 (13.3%) patients out of 95			
Minor neurologic d​eficits (ND)	13 (12.4%) patients out of 95			

This together with the reasonable adverse events (AE) reported, the outcomes they obtained are comparable to those reported by other investigators. Karlsson, et al. obtained similar results in 2007 [18]; they used controlled doses of nBCA to embolize 18 patients with large AVM before radiosurgery and achieved 68% obliteration rate. Additionally, Gobin, et al. in 1996 [25] treated 125 patients harboring massive AVM volumes, the majority of them ranged from SM-III-VI. The combined treatment strategy was to use nBCA to perform embolization before radiosurgery by GK. Their embolization results showed 11.2% total occlusion cases, 76% reduction in AVM sizes, making them small enough to favor radiosurgery. Subsequently, they reported outcomes of 59% (53 out of 90 patients) obliteration rate, 12.8% morbidity, 1.6% mortality, and three percent hemorrhage. The operation was performed successfully with revascularization occurring in only 11.8% of the total number of patients within a year. Therefore, Dalyai and co-workers concluded that this combined approach is very useful for the treatment of large and complex AVMs [14]. Several investigators have also obtained improved obliteration outcomes with combined treatment, ranging between 50%-78% with minimal SAEs [17,25,37-39], as displayed in Table 4.

### Timing after AVM treatment is critical in determining outcome

**Figure 3:**
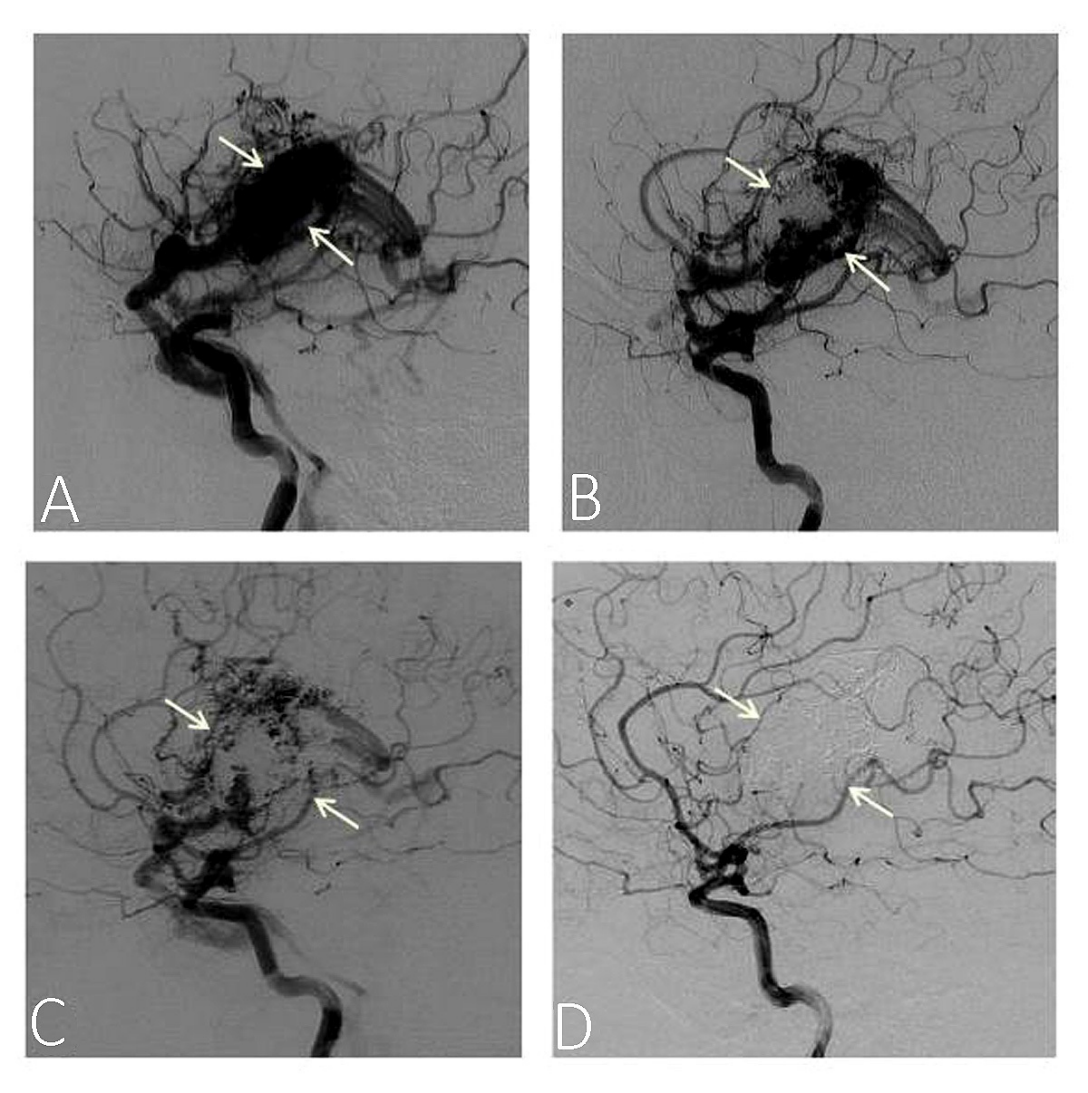
Angiograms showing the time course of nidus obliteration rate in AVM patient from zero to five years Onyx treatment sessions were performed for four years and combined Onyx/stereotactic radiosurgery (SRS) treatment occurred in the fifth year. Selected panels from Pierot and his co-workers study, the left internal carotid digital subtractive angiograms (DSA) shows the time course of nidus obliteration of AVM patient, before treatment (A) after two years of two Onyx treatment sessions (B), Four years after treatment sessions showing only residual nidus (C) and five years after having a combined fifth Onyx session and SRS (D). Complete obliteration of the nidus occurred. White arrows indicate the position of the nidus. Figure reproduced from Pierot, et al. [17]

In the Pierot, et al. report of 2013 [17], they performed series of Onyx sessions, followed by combined Onyx-SRS treatment of the residual nidus, on 20 AVM patients between 2003 and 2008. The AVMs ranged from five patients with SM I-II, 10 with SM III-IV and five patients with SM V, with a follow-up period of two-five years. By five years, 17 patients remained; five out of seven with SM I-II attained 71.4 % complete obliteration, five out of 10 with SM III-IV reached 50%. The cumulative obliteration rate recorded was 58.8% with minimal adverse effects of five percent. One interesting result showed by these authors was the revelation of gradual reduction of the size of the nidus of a patient over a time course. Figure 3 shows the left internal carotid DSA before (Figure 3A) and after treatments (Figures 3C-3D).

By the second year, about half of the nidus had obliterated (Figure 3B); in the fourth year, there was still some residual nidus (Figure 3C), complete obliteration occurred in the fifth year (Figure 3D), during which the patient underwent a fifth and final Onyx session, followed by SRS procedure. These results suggest that timing is an important factor that can influence the outcome and contribute to the controversial reports observed in various studies and that over time, more patients could have a successful recovery from this treatment strategy.

### Combined AVM treatment diminishes the quality of outcomes in certain instances

In Kano, et al. 2012 paper [37], 120 AVM patients underwent two-three embolization procedures before gamma knife surgery (GKS) or had only GKS treatment. A previous history showed that 64 patients had experienced at least one previous hemorrhage. Target AVM volume ranged between 0.2-26.3 cm^3^, with a median of 6.6 cm^3 ^and median SM grade of III. Radioactive dosage median was 18 Gy. There was 19 years follow-up period (1987-2006). Percent obliteration was determined by MRI imaging or angiography images with subsequent data analysis. Hemorrhage, neurological deficits due to adverse radiation effects (ARE) and other parameters assessed. Summarized in Table 3 is a comparison of results for the single versus combined treatments for two and four-year outcomes.

**Table 3:** Summary of outcomes of AVM patients treated with single stereotactic radiosurgery (SRS) or combined embolization (embo)/SRS over four-year follow-up period

Characteristics	Embo/SRS	SRS
Total obliteration rate at four years	53%	71%
Hemorrhage rate after SRS at two years	3.5%	3.4%
# of Symptomatic AREs	11	10

The authors observed that patients with smaller AVM volumes had better success, as compared to those with large masses and that disappointingly, there was the overall reduction in the cumulative total obliteration rate with the combined treatment (53%) than single (71%). Similarly, several groups have shown a parallel trend of results [37-39]. Sirin, et al. [38] in 2006, studied outcomes in 28 patients with gigantic AVM sizes (SM IV-VI), after embo/SRS treatment and obtained obliteration rates as worse as 20%-29% over three years follow-up period. Lee, et al. in 2015 [39], studied 199 AVM patients with a subsequent four-year follow-up. The obliteration rate with combined (Onyx/SRS) treatment was 24%, and the single SRS was 40%. Koga, et al. in 2011 [30] obtained reasonable obliteration rate of 50% with 44 patients but obtained a poor casualty rate of 12%. Xu, et al. [34] carried out cohort studies on 1,988 AVM patients and attained worse obliteration results with the combined (41%) than the single SRS (59%) treatments. Finally, Ding, et al. [26] recently reported that repetitive treatments of giant size AVMs with SRS yielded poorer obliteration outcomes of 67%, 36%, and 73% at zero, three, and 10 years respectively and worse SAEs. By contrast, single SRS treatment generated better obliteration rates of 79%, 53%, and 84% over the same time course with less SAEs (Table 4).

**Table 4:** (N) Number of patients, (^0-1 yr,*1-3 yrs; **4-6 yrs; ***7-10 yrs), follow-up periods, (NR) Not recorded, (ND) Not determined, (P-ND) Permanent neurological deficits, (S) Values for single SRS, (R) Values for repeated SRS, (m) Mean, (Es) Estimated from the angiographies).

Authors	N	SM Grade	% Obliteration SRS Embo Embo/SRS	% Hemorrhage	% P-ND	% Mortality
Obliteration rate increased with combined Embo/SRS treatment						
Beltramello et al. (2005) [29 ]	113 98	I-11 III-V	NR ND ND ND 28.6^ >50**	NR 2	NR 7	NR 0
Daylai et al. (2014) [14]	95	III-V	40 0 45*, 56**,63***	8	5	0
Ding et al. (2013) [15]	148	III-V	NR 0 68***	1.6	2	0
Pierot et al. (2013) [17]	20	I-II III-IV	NR NR 71.4** NR NR 58**	0	10	0
Pollock et al. (1996 b) [20]	34	IV-V	NR NR 65**	2.4	0	0
Gobin et al. (1996) [25]	125	II-VI	NR NR 65*	3	0	1.6
Blackburn et al. (2011) [31]	21	IV-V	NR NR 81*	0	0	0
Han et al. (2013) [32]	28 10	I-II III-IV	100** ND ND ND NR 73**	1.5	13.8	6.9
Zabel–du Bois et al. (2007) [33]	50	II-1V	ND ND 67*, 78**	2.2	NR	0
Obliteration rate was worse with combined Embo/SRS or repeated SRS treatment						
Kano et al. (2012) [37]	120	I-VI	ND NR 35*, 55**, 59***	2.7	2.5	5.8
Sirin et al. (2006) [38]	28	IV-VI	ND NR 21-29*	2.7	5-14	7
Koga et al. (2011) [30]	44	II-IV	ND NR 52**	2.4	5	12
Xu et al. (2014) [34]	1988	II-VI	59* NR 41*	7.3	3.3	0
Ding et al. (2016) [26]	554 84	I-V I-V	S=79^, 53*74**, 84*** R=67^^^, 36*, 57**, 73***	3.8 (m) 10 (m)	7 (m) 13 (m)	1.2 1.2
Obliteration rate did not change significantly with combined Embo/SRS treatment						
Wang et al. (2014) [35]	116	IV-VI	82** NR 82**	1.9	0.8	0
Sousa et al. (2016) [36]	59 31	III-V	95*(Es) ND ND ND 95* (Es)	2.2	6.7	0
Karlsson et al (2007) [18]	133	III-IV	62 ND ND	7	7	0
Maruyama et al. (2005) [40]	32	II-V	64*, 72**ND ND	0	3	0

This finding provided awareness that successive SRS treatment of giant size AVMs is not an ideal strategy for treatment, in particular, if they localize in eloquent brain regions and accounts for the reason why the need of good embo materials is crucial for the reduction of large nidus sizes prior to SRS.

### It is possible to combine embolization and SRS in AVM treatment without appreciable change in outcome

Wang, et al. in 2014 [35] used the same approach on patient groups (SM 1-II, SMIII-IV, SM V-VI) and treatment techniques and showed remarkable percent obliteration score (81.9%) for both SRS alone or combined embo/SRS treatments in 116 patients with minimal SAEs. Sousa, et al. [36] gathered similar trend of results. Although their data did not quantitate the obliteration rates, deductions made from the angiography are that of complete obliteration occurred with both types of treatments. Additionally, Karlsson, et al. [18], Sousa, et al. [36] and Maruyama, et al. [40] obtained reasonably high obliteration rates between 64%-72% with large AVMs after SRS alone treatments (Table 4). Further, Mortazavi, et al. [41] carried out parallel studies in 2013 using the similar techniques and methods and showed no significant difference in obliteration outcomes and adverse events over three-year follow-up period with combined treatments. Finally, Henkes, et al. in 1998 [42] reported complications such as persistent venous stagnation and intranidal aneurysm encountered in combined embolization and SRS therapy and arrived at a conclusion that it is rare to achieve an overall fruitful outcome with combined treatments.

### Plausible factors account for setbacks in AVM treatment outcomes

This review sought to use different case-oriented studies to show data from single as well as combined AVM treatment outcomes and how these results had been very diverse. A particular view of the literature has revealed several reasons why inconsistencies in outcomes reported by different groups should not be surprising. We determined significant bias introduction into the various studies from different clinical settings, which we have indicated below as crucial factors that require consideration when planning AVM surgery:

1) AVM size and complexity determines the aggressiveness of treatment [9,14,17]. Therefore, it is important to have the precise measurement of sizes; size should also be a critical determinant of grouping patients for treatment to minimize variations in outcome.

2) AVM drainage into the deep venous system is a crucial determinant of neurological deficits; therefore, it is important to aim at preventing disruption of the AVM surroundings, as this can impair drainage and cause frequent hemorrhage [9,16].

3) The nature of adjuvant used in embolization is essential, as the liquid types, such as polyvinyl alcohol (PVA) and nBCA are leaky and can cause distal catheter gluing during surgery [17,29]. In fact, studies have revealed that embo complications can be quite dramatic; accounting for over 10% morbidity and mortality rates [27,42]. The use of Onyx, a copolymer, however, has an advantage of precipitating blood upon contact, avoiding gluing and thus has become a more favorable embolizing material [43-44]. Additionally, Onyx can occlude large portions of the nidus through series of injections to prevent hemorrhage and will reduce the AVM size by at least 50% for accessibility and safety during SRS [45].

4) Staging the dosage of an embolization material per subgroup is an important determinant for an effective therapy and requires careful calculation. Under dosage will not work efficaciously and ultimately results in a reduction in obliteration rates; and over dosage can cause SAEs such as speech and vision impairment, partial paralysis, seizures and even increased mortality rates [2,28].

5) The depth and cerebral localization of the AVM is an important determinant of treatment strategy. If an AVM is in the non-eloquent region of the brain and not deeply embedded, the catheter can reach the nidus for both embolization and SRS procedures to a favorable treatment outcome. A giant AVM in the deep eloquent region, however, will either, a) presents itself as embolization and SRS resistant spot, or b) an attempt to treat it could result in a deleterious adverse event (AE) such as increased intracranial hemorrhage, loss of speech, vision, and stroke [2,36]. For AVMs in such obscure and sensitive regions, there is the need to use much thinner catheters and microwires to be able to direct them into the nidus, and also a careful balance between health and risk decision need to be made about radioactive dosage to make the combined treatment much safer and feasible.

6) After performing embolization, recanalization of the nidus can occur in such a way that the blood vessel lumen of the occluded feeders to the nidus is restored spontaneously [29] and it can bring about re-expansion and change in its configuration, thereby, limiting access to the SRS procedure.

7) Liquid embolization materials such as nBCA and ethylene-vinyl alcohol copolymer (EVOH) can cause beam attenuation during GSK procedures such that the radiation dose (the number of kilovoltage radiation photons) reduces in an embolized nidus [46-47]. Onyx, which has been shown to have profound embolization advantages [17,44-45], unfortunately, was reported in a few studies that it could also cause a reduction in beam dosage [17,46-48]. Consequently, this can reduce obliteration rates and have a negative impact on outcomes. Fortunately, if such technical limitation is observable at initial treatment stages, adjustments could be made for a patient to overcome the effect. it is wise to increase the radiation dosage performed by employing a high-powered (60) Co beam with higher radiation strength [47].

8) Aneurysms are present in the arteries feeding the nidus and can rupture because of their delicate nature and cause bleeding in the AVM region [49]. SRS procedure can delay because the neurosurgeon has to use a microscope to isolate the blood vessel that feeds an aneurysm and halt its blood supply to enable the SRS procedure performed. Consequently, this can bring about hemorrhagic stroke.

9) Individuals diagnosed with AVM are at risk of suffering from intracranial hemorrhage, seizures, vascular diseases, neurological deficits, and stroke making it difficult for drastic surgery decisions for them. Therefore, pre-existence of any of these conditions prior to AVM diagnosis could result in higher risk of experiencing AVM progression into maturation [18-19], or if the surgeons would be compelled to operate on the existing AVM, the patients are likely to undergo SAEs. Because of this plausible sensitivity of patients’ condition, a much more invasive treatment decisions are difficult to make [50] and so poor obliteration outcomes would be the consequence.

## Conclusions

Our analysis set to determine reasons for inconsistencies in AVM combined embolization and SRS treatment outcomes. Certainly in a cohort study, if a subset of patients in a group encounters any of these nine setbacks mentioned above, it will influence a cumulative outcome. Our analysis clearly provides an awareness that the reported varying outcomes in the treatment of patients bearing giant size AVMs might be due to lack of blending all of these crucial factors when planning individuals’ customized treatments. Recent advances in endovascular techniques and discovery of better embolizing adjuvants should aid in minimizing these contradictory reports and bring about more reliable, efficient and safer operations.
